# An Intact *Ex Vivo* Pigskin Model for Evaluating Topical Treatments for Blepharitis‐Associated Biofilms

**DOI:** 10.1155/joph/5599593

**Published:** 2026-07-29

**Authors:** Aaron Strickland, James Gras, Ellen Lantz, Richard Eiferman, Anthony Sawyer, George Rodeheaver

**Affiliations:** ^1^ iFyber, Ithaca, New York, USA; ^2^ Department of Ophthalmology, University of Louisville, Louisville, Kentucky, USA, louisville.edu; ^3^ Nevada Naturals, Inc., Queen Creek, Arizona, USA; ^4^ Department of Plastic Surgery, University of Virginia Health Sciences Center, Charlottesville, Virginia, USA, virginia.edu

## Abstract

**Importance:**

Blepharitis is a chronic inflammatory condition of the eyelid margin that impacts millions of adults and children. Its etiology is unclear, and its treatment remains unsatisfactory.

**Objective:**

To develop an intact *ex vivo* pigskin model to evaluate the etiology of and to determine effective treatments for blepharitis.

**Design:**

Mature bacterial biofilms of *Staphylococcus epidermidis* were generated on intact pigskin explants. These biofilms were treated topically for 24 h before quantifying the residual biofilm bacteria.

**Results:**

The amount of total bacteria after treatment with phosphate buffered saline (PBS) was 7.29 log. Using common clinical treatments, the amount of bacteria was 5.05 log for 10 % povidone iodine (*p* < 0.05) and 4.96 log for 0.01% HOCl (*p* < 0.05). The amount of bacteria for gentamicin treatment was 6.38 log, which was not significant from phosphate buffered saline (PBS) treatment (*p* = NS). Using a prototype formulation, the level of bacteria was reduced to a non detectable level which was significantly lower than all other treatments (*p* < 0.05).

**Conclusions and Relevance:**

A model for studying bacterial biofilms on intact *ex vivo* pigskin explants was developed. Using this model, it was shown that a prototype formulation containing naturally derived antimicrobials and a biofilm disrupter was superior to several commonly used clinical treatments in eliminating biofilm bacteria. We believe this effective, antibiofilm formulation can be used safely in the eye for clinical applications.

## 1. Introduction

Blepharitis is a chronic inflammatory condition of the eyelid margin [1]. It typically presents with redness, swelling, and irritation of the eyelids. If the cornea is involved, blepharitis can cause blurred vision and photophobia. Blepharitis is also one of the most common ocular pathologies encountered in clinical settings, impacting millions of adults and children. Its etiology remains unresolved but is likely multifactorial. Because the specific etiology of blepharitis has not been elucidated, numerous therapeutic modalities have been proposed; however, they do not exhibit lasting results [2–4].

Many clinicians believe that enhanced bacterial bioburden is a primary causative agent in the etiology of blepharitis [5–8]. One of the most prevalent bacteria associated with this etiology is the coagulase‐negative *Staphylococcus epidermidis* [6, 7]. Therefore, oral or topical antibiotics are often prescribed as a treatment option for blepharitis [2, 3, 9–11]. However, antibiotics usually provide only short‐term benefits. These limited results need to be balanced against the possibility of developing or selecting antibiotic‐resistant strains.

Recent studies of chronic inflammatory diseases have suggested that the causative agent may be related to bacterial biofilms [12, 13]. In 2016, Rynerson and Perry [14] proposed that blepharitis occurred due to bacterial biofilm formation. The difficulty in eradicating biofilms with antimicrobial agents and immune mechanisms could explain why existing treatment regimens are ineffective against the primary problem.

Newer treatments should focus on the disruption and eradication of the causative biofilms. In this study, we used full‐thickness porcine skin as an *ex vivo* model [15] to study the efficacy of a novel treatment formulation to disrupt and eradicate established *S. epidermidis* biofilms. Porcine skin is considered a suitable substitute for human skin due to its anatomical, physiological, and biochemical similarities, making it valuable for dermatological research, wound healing studies, and cosmetic or pharmaceutical evaluations. In particular, the thickness of the epidermis closely resembles that of human skin. Both have stratified squamous epithelium with similar collagen and elastin distribution and density. Porcine skin also has hair follicles and sebaceous glands [16], making it useful for testing inflammatory skin conditions such as blepharitis or eczema.

## 2. Materials and Methods

### 2.1. Bacterial Strains and Culture Conditions

*Staphylococcus epidermidis* (ATCC 35984) was used in this study. Overnight cultures were prepared in tryptic soy broth (TSB) for 18–24 h. Log‐phase cultures were prepared on the same day of conducting the assay by preparing a subculture in TSB at 37°C and > 80% relative humidity. The subculture was used for inoculation when the optical density at 650 nm (OD_650_) was between 0.2 and 0.4, corresponding to approximately 10^6^–10^7^ colony‐forming units per milliliter (CFU/mL). These subcultures were used to inoculate the porcine skin explants, as detailed in the following.

### 2.2. Preparation of Porcine Skin Explants for Testing

Quality control: Full‐thickness porcine dermal tissue with an intact epidermis was sourced from Sustainable Swine Resources (Sheboygan Falls, WI, USA), an ISO‐22442‐2‐compliant facility. Tissue was supplied in approximately 10 × 10 cm sheets, and the incoming bioburden was assessed according to an iFyber standard operating procedure (SOP). In brief, the tissues were inspected for physical damage (such as scalding, irritation, and cuts/scratches) and assessed for markings on the tissue surfaces (ink/paint) from the processing facility, which were areas that were avoided for experimentation. Representative areas of the skin (*n* ≥ 4) were assessed for thickness and excess hair length, and the tissue was discarded if the thickness and hair length were outside the following specifications: 3–4 mm thickness and hair length > 2 mm. The tissues were then cut into 5 × 8 cm sections for storage at −80°C. Finally, the incoming bioburden of the tissue was assessed by cutting multiple 0.5‐inch‐diameter punch biopsies of the tissue (*n* ≥ 6) under aseptic conditions. The biopsies were then transferred to sterile tubes, and the bacteria were recovered via sonication in phosphate buffered saline (PBS) containing 5 ppm Tween 80 and plated on tryptic soy agar (TSA). The tissue lot passed inspection if the average incoming bioburden was ≤ 4 log CFU per biopsy.

Sterilization: The tissue used for experiments was sterilized at NovaSterilis, Inc. (Lansing, NY) using a supercritical carbon dioxide (scCO_2_)–based sterilization process and a liquid sterilant referred to as NovaKill, which is a mixture of water, peracetic acid (PAA), and hydrogen peroxide. This sterilization process has been validated at a sterility assurance level of 10^−6^. In brief, tissue samples were sterilized in batch sizes of 24, resulting in no microbial growth after neutralizing the residual peroxides in dilute sodium thiosulfate (6 g/L) and incubating in TSB for up to 2 weeks. After the sterilization cycle (scCO_2_ pressure of 1380 psi for 90 min), the tissue appeared much lighter in color, which was likely due to the removal of residual blood components. Treatment of the sterile tissue with sodium thiosulfate resulted in PAA levels that were below the detection limits (e.g., 0.5 ppm) using a NovaPak (NovaSterilis), which utilizes a colorimetric assay similar to that described in the literature [17]. We found that sterilized tissue treated with thiosulfate can either be used immediately to culture biofilms or stored for up to 1 month at −80°C with no change.

### 2.3. Porcine Skin Inoculation and *Ex Vivo* Biofilm Formation

This study utilized an *ex vivo* porcine dermal model of mature biofilm following previously published work by Phillips and co‐workers [18]. Rather than creating artificial “wounds” in the explants through the site‐specific removal of the epidermis, this study utilized porcine skin that had an intact epidermis and dermis. Mature biofilms were then established on the apical side of the intact skin explants measuring 2.5 × 2.5 cm by inoculating the explants with *S. epidermidis* (ATCC 35984) subcultures with an OD_650_ of 0.2–0.4. Three techniques were employed to spread the inoculum reproducibly across the porcine skin surface: (1) A cotton swab saturated with a 10^6^–10^7^ CFU/mL inoculum, (2) an inoculating loop to spread 50 μL of a 10^6^–10^7^ CFU/mL inoculum, and (3) a 4‐ply nonwoven Curity gauze (Covidien, Dublin, IE) that was cut to the size of the explant and saturated with 10^6^–10^7^ CFU/mL of an *S. epidermidis* subculture. A sterile glass slide was placed on the top of the gauze pad to ensure even contact between the gauze and tissue surface. After 2.5 h, the gauze and glass slides were removed. All tissue samples were then transferred to plain 0.5% agar plates to maintain hydration and incubated in a stationary incubator at 37°C and > 80% relative humidity. Tissue samples were transferred to fresh 0.5% agar plates daily for 72 h.

### 2.4. Establishing the Presence of Mature Biofilms on Intact Porcine Skin Explants

The resulting inoculated porcine skin explants were assessed for the presence of biofilms according to iFyber’s SOPs. Because no reliable biological markers of biofilm formation exist, we described a unique phenotype of bacteria on the intact skin explants tolerant to hypochlorous acid (HOCl) concentrations above the minimum bactericidal concentration for planktonic *S. epidermidis*, which was determined using standard methods to be ≥ 0.1% HOCl in 400 mM phosphate buffer at pH 5.8. Biofilm formation was confirmed on replicate explants (*n* = 3) using CFU plate counts.

CFU counts: After incubating the inoculated explants for 3 days, as described above, samples were washed with PBS to remove adventitiously bound debris and bacteria and subjected to differential detection of biofilm‐associated bacteria using punch biopsies. In brief, 0.8‐cm‐diameter punch biopsies were excised from the four corners of each of the three replicate tissue samples for CFU counts. Two punch biopsies from each replicate sample were transferred to sterile tubes and washed in Dey‐Engley broth (D/E) to determine the total bacterial count (planktonic plus biofilm). The remaining two were fully immersed in 0.1% HOCl for 20 min to kill only planktonic bacteria. All biopsies were then washed with D/E, and the remaining bacteria were recovered in D/E via sonication, enumerated using standard 10‐fold serial dilutions, and plated on TSA.

### 2.5. Treatment of Explants Containing 72‐h *S. epidermidis* Biofilms With Antimicrobial Agents

Tissue samples (2.5 × 2.5 cm) containing 72‐h mature *S. epidermidis* biofilms were washed with PBS to remove adventitiously bound debris and bacteria. No exposure to 0.1% HOCl was performed. We then tested the efficacy of commonly used antimicrobial agents to eradicate mature biofilm bacteria: PBS (as a control), 10% dilute povidone iodine (Betadine®) used clinically in surgical skin preparation (diluted in PBS), an aqueous antibiotic solution (3 mg/mL gentamicin), a commercial product (Avenova®) used in the treatment of eyelid infections (0.01% HOCl), and an experimental liquid treatment formulation that combined natural fatty acid esters (monolaurin, sucrose monolaurate, and lauroyl arginine ethyl ester) with a surfactant (Poloxamer 188). Five explants were used for each treatment.

In brief, explants were treated for 24 h by immersing them in 6 mL of each formulation at 37°C and > 80% relative humidity in individual 60 × 15 mm Petri dishes. After 24 h, the treatments were aspirated, and the explants were washed three times in 6 mL PBS (2 min soak each time), and the total number of viable bacteria was recovered from the tissue using an ASTM cup scrub method in D/E broth [19]. This method was more efficient than using individual biopsies. This method is a modified lavage wash that utilizes an open polypropylene cylinder (15 × 15 mm) and a polypropylene policeman scraping tool. The cylinder was centered on top of the tissue sample and pressed tightly onto the pigskin explant while 1 mL of D/E solution was added. The policeman tool was used to reproducibly scrape and lift the viable bacteria into the Tween 80 solution. The resulting bacterial solution was then sonicated and vortexed, and enumeration was performed using standard 10‐fold serial dilution and plating on TSA plates.

### 2.6. Statistical Analysis

Data for experiments assessing the efficacy of biofilm removal were compared statistically using a one‐way analysis of variance (ANOVA). When a statistical difference was detected, Tukey’s post hoc multiple‐comparison test was used to identify differences between individual groups, where a *p* value of < 0.05 was considered significant.

## 3. Results

### 3.1. Establishment of an *S. epidermidis* (ATCC 35984) 72‐h Biofilm on Intact, Sterilized Porcine Skin

**FIGURE 1:**
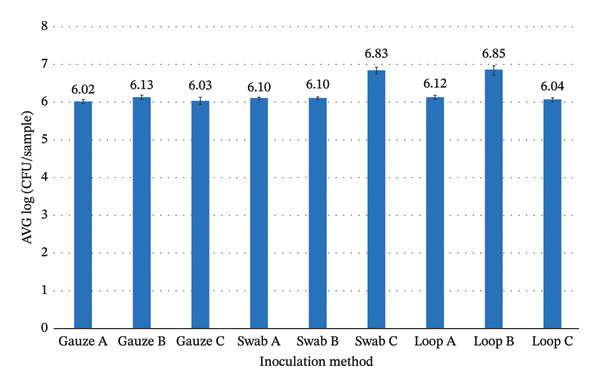
Each technique used to inoculate the surface of the sterile porcine skin produced a 72‐h total bioburden of log 6.02–6.83 CFU/explant. The easiest and most consistent technique involved using a soaked gauze overlay. This technique was used for all studies.

We found that the most consistent method for inoculating the sterilized porcine skin for biofilm formation was using a gauze soaked in an early log‐phase subculture of *S. epidermidis*. While all inoculation methods resulted in an average total microbial loading of 6 log CFU/explant (biofilm and planktonic) after 72 h, the gauze technique was most convenient and consistent relative to the other methods (Figure 1).

### 3.2. Differential Detection of Biofilm vs. Total Microbial Load of 72‐h *S. epidermidis* Biofilms on Intact Porcine Skin

**FIGURE 2:**
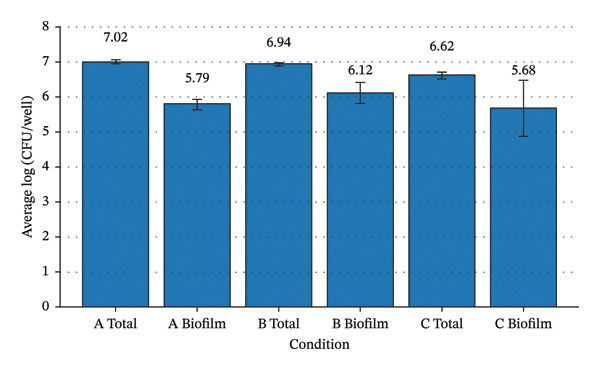
The fraction of the total bioburden associated with biofilms can be differentiated by treating half of the samples with HOCl (0.1%), which kills the planktonic fraction. A, B, and C represent individual explants containing 72‐h biofilms. From each explant, 2 biopsies were counted for total bacteria and 2 for biofilm‐only bacteria. The average (*n* = 6) total microbial load was 6.86 log colony‐forming units (CFU)/explant, and the average (*n* = 6) biofilm fraction was 5.87 log CFU/explant, indicating an approximate differential of 1 log CFU/explant of planktonic bacteria compared to biofilm‐related bacteria.

After using the gauze technique for establishing a 72‐h biofilm on the porcine skin explants, we determined the fractional amount of *S. epidermidis* in the biofilm phenotype vs. total bacteria (i.e., the planktonic and biofilm phenotypes). We observed an average total bacterial load of 7.29 log CFU/explant and an average biofilm fraction of 6.87 log CFU/explant, indicating an approximately 1 log CFU/explant reduction in the proportion of planktonic bacteria relative to those in biofilms (Figure 2).

### 3.3. Use of the Mature Biofilm Model on Intact Porcine Skin to Assess the Efficacy of Commercial and Prototype Skin Treatments Against *S. epidermidis* Biofilms

**FIGURE 3:**
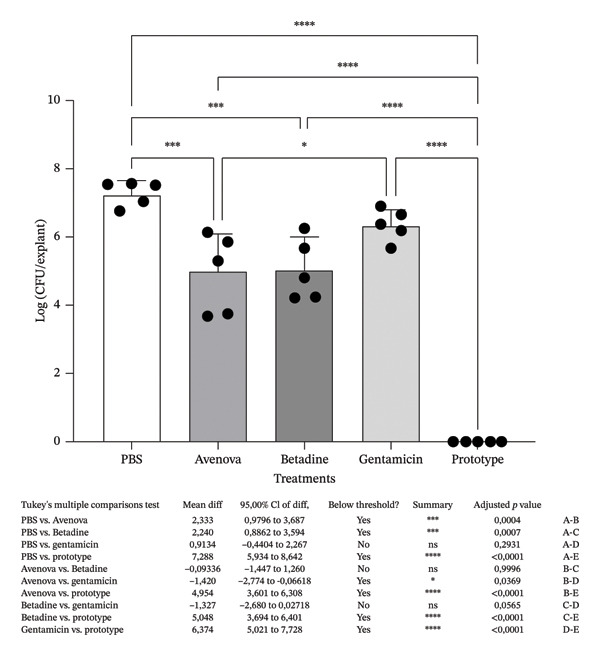
All treatments except gentamicin significantly (*p* ≤ 0.05) reduced the total number of bacteria compared to the PBS‐treated controls. The most effective agent in reducing the total number of *S*. *epidermidis* bacteria was the prototype agent. This agent was significantly superior (*p* < 0.05) in eradicating bacteria compared to the other antimicrobial agents tested. Data are presented as mean ± standard deviation (SD) from five independent replicates (*n* = 5).

In addition to the experiments described above for HOCl, explants with mature 3‐day cultures of *S. epidermidis* containing approximately 6 log of HOCl‐tolerant biofilms were also treated with various commercial and prototype treatments for skin infections (Figure 3). Data are presented as mean ± one standard deviation (SD) for five independent replicates. The differences observed for the bacterial count (CFU/explant) of 24‐h treated explants were statistically significant (*p* < 0.05) for all treatments relative to the PBS control except for gentamicin. A 10% dilution of povidone iodine (Betadine) in PBS showed an approximately 2.24 log reduction. Therapeutically relevant concentrations of gentamicin (3 mg/mL) showed only an approximately 0.91 log reduction relative to the PBS‐treated control and were not significantly different. Avenova, a clinically utilized treatment, showed a 2.33 log reduction in DFUs compared to PBS, which was significantly different. The experimental prototype formulation comprising a nonionic surfactant combined with a dilute mixture of all‐natural fatty acid esters showed a greater than 7‐log reduction in the amount of biofilm bacteria relative to the PBS‐treated control. Collectively, these results highlight the robustness of the biofilm produced on the intact porcine skin. Moreover, while some commercial skin treatments and therapeutically relevant levels of antibiotics can reduce the biofilm load attached to the porcine skin, only the novel experimental treatment formulation exhibited a 7‐log reduction relative to the controls.

## 4. Discussion

Blepharitis is a chronic inflammatory disease of the eyelids whose pathogenesis is not completely understood. Many clinicians believe that bacteria play an important role in the etiology of blepharitis. One bacterium commonly associated with the disease is *S*. *epidermidis* [6, 9]. A recent report stated that this organism exists primarily within biofilms [14].

Biofilms are a complex community of microbes attached to a surface and bound by an extracellular matrix (ECM). This ECM is composed predominantly of polysaccharides, proteins, and DNA fragments. The ECM protects the bacteria from the body’s immune system and external biocides, including antibiotics. Biofilm bacteria are considered tolerant to antibiotics because their metabolic activities are significantly reduced [20]. Their antibiotic tolerance may be as much as 10–1000 times greater than their sensitivity in the planktonic phenotype. With this defensive matrix, blepharitis biofilms would not be susceptible to standard clinical treatments, which is the reality for clinical cases of blepharitis.

To improve the treatment of blepharitis, having a reproducible ex vivo model to evaluate various therapeutic approaches would be beneficial. This report describes the development of a biofilm model using full‐thickness pigskin with intact dermis and epidermis. Pigskin is anatomically similar to human skin. Of importance is the presence of a complete hair follicle with its associated sebaceous gland [16]. These structures adequately simulate the eyelid and eyelashes. The model used an overnight culture of *S. epidermidis* to uniformly cover the surface of sterilized pigskin patches (2.5 × 2.5 cm). After 72 h of incubation, the presence of a mature biofilm was confirmed by quantitating the number of *S. epidermidis*, which we established as a mixture of planktonic and biofilm‐associated bacteria. The biofilm fraction contained an average of 5.87 log CFU/explant. Using this model, it was shown that biofilm bacteria are difficult to eradicate with clinically utilized agents. Antiseptics such as povidone iodine and HOCl did not effectively eradicate the *S. epidermidis* within the biofilm matrix. Even the antibiotic gentamicin was ineffective against the antibiotic‐tolerant bacteria within the biofilm matrix.

The prototype formulation using a biofilm‐disrupting agent and an antimicrobial mixture effectively eradicated all bacteria from biofilm‐coated intact skin explants [21–23]. To improve the efficacy of antimicrobials and the immune system in eradicating biofilm bacteria, it would be beneficial to disrupt the biofilm matrix. However, the rapid dispersal of the biofilm matrix may have clinical consequences. The burst of high levels of planktonic bacteria from a biofilm matrix can overwhelm the body’s defense systems and lead to increased local infection or, in extreme cases, may lead to septicemia and even death [24]. Hence, using a matrix‐disrupting agent concurrently with an antimicrobial agent would be prudent for treating biofilm bacteria.

The results of this study document that a synergistic combination of naturally derived Generally Recognized as Safe (GRAS) antimicrobial fatty acid esters and a surfactant significantly reduced the biofilm‐sequestered bacteria. Our therapeutic prototype formulation was derived from lauric acid. Lauric acid, a C12 medium‐chain fatty acid, makes up 45%–53% of coconut oil [25]. The esterification of lauric acid with glycerol produces monolaurin, a potent antimicrobial agent [26]. In this study, monolaurin was synergistically combined with sucrose monolaurate and lauroyl arginine ethyl ester, a widely used food preservative in ready‐to‐eat meats, to create an effective antimicrobial formulation. When tested in an ex vivo porcine skin model containing a partial‐thickness wound [27], these fatty acid esters achieved clinically relevant reductions of mature *Pseudomonas* bacterial biofilm within 24 h of exposure. [28–30]. When tested on the ex vivo intact porcine skin model described in this report, the results were similar when tested against *S. epidermidis* biofilms. The prototype formulation eradicated the mature *S. epidermidis* biofilm. Furthermore, the surfactant used in the test formulation was Poloxamer 188. Poloxamer 188 is nontoxic to cells [31] and is classified as GRAS by the FDA. This linear block copolymer of polyoxyethylene and polyoxypropylene has a hydrophobic core with hydrophilic termini. The amphipathic structure of Poloxamer 188 can interact with the components of the biofilm matrix and cause bacterial cell detachment and dispersal. Several reports have documented this in vitro [32, 33], ex vivo [34], and in vivo [35, 36]. Upon biofilm matrix dispersion, the released planktonic bacteria become susceptible to antimicrobial agents [35–37].

## 5. Conclusion

Blepharitis is a chronic, inflammatory disease of the eyelid. Recent reports suggest that the etiology of this disease involves bacterial biofilms, with the most prevalent bacterial component being *S. epidermidis*. Herein, we report the generation of a reproducible, *ex vivo* model of mature *S. epidermidis* biofilms on excised, intact porcine skin. This model was used to evaluate the antimicrobial efficacy of several clinically relevant antimicrobial agents, which were shown to be ineffective. However, a novel prototype formulation of naturally occurring antimicrobial esters and a surfactant was shown to be significantly superior to the other agents tested in reducing the number of biofilm bacteria in the porcine skin model. This prototype formulation is completely biocompatible and may be easily applied to the eyelid to effectively treat blepharitis.

## 6. Limitations

This study demonstrates that a prototype formulation containing a biofilm disruptor is significantly more effective than using antimicrobial agents alone. One limitation of this study is that the prototype formulation has not been optimized. Another limitation is that histological analysis has to be conducted to prove that the biofilm at the base of the hair shaft has been eradicated using the biofilm disruptor. Future research may require an additional agent to enhance the penetration of the prototype formulation to the base of the hair shaft.

This study postulates that the etiology of blepharitis is associated with the formation of bacterial biofilm. This biofilm formation restricts the egress of meibum, resulting in meibomian gland dysfunction (MGD). A limitation of this study may be that MGD may be caused by factors other than biofilm. If that were the case, the intact pigskin model may still be useful to study other inflammatory processes within the hair follicle.

This study was conducted with an ATCC strain of *S. epidermidis.* Once the prototype formulation has been optimized, another study should be performed to prove that the optimized formulation is effective against several clinical strains of *S. epidermidis* obtained from patients with blepharitis.

## Funding

No funding was received for this study.

## Conflicts of Interest

The authors declare no conflicts of interest.

## Data Availability

The data that support the findings of this study are available on request from the corresponding author. The data are not publicly available due to privacy or ethical restrictions.
